# Visual Network Asymmetry and Default Mode Network Function in ADHD: An fMRI Study

**DOI:** 10.3389/fpsyt.2014.00081

**Published:** 2014-07-15

**Authors:** T. Sigi Hale, Andrea M. Kane, Olivia Kaminsky, Kelly L. Tung, Joshua F. Wiley, James J. McGough, Sandra K. Loo, Jonas T. Kaplan

**Affiliations:** ^1^Department of Psychiatry and Biobehavioral Sciences, UCLA Semel Institute for Neuroscience and Human Behavior, Los Angeles, CA, USA; ^2^Department of Psychology, University of California Los Angeles, Los Angeles, CA, USA; ^3^Department of Psychology, Brain and Creativity Institute, University of Southern California, Los Angeles, CA, USA

**Keywords:** attention, laterality, asymmetry, sensory, verbal, default, spatial, network

## Abstract

**Background:** A growing body of research has identified abnormal visual information processing in attention-deficit hyperactivity disorder (ADHD). In particular, slow processing speed and increased reliance on visuo-perceptual strategies have become evident.

**Objective:** The current study used recently developed fMRI methods to replicate and further examine abnormal rightward biased visual information processing in ADHD and to further characterize the nature of this effect; we tested its association with several large-scale distributed network systems.

**Method:** We examined fMRI BOLD response during letter and location judgment tasks, and directly assessed visual network asymmetry and its association with large-scale networks using both a voxelwise and an averaged signal approach.

**Results:** Initial within-group analyses revealed a pattern of left-lateralized visual cortical activity in controls but right-lateralized visual cortical activity in ADHD children. Direct analyses of visual network asymmetry confirmed atypical rightward bias in ADHD children compared to controls. This ADHD characteristic was atypically associated with reduced activation across several extra-visual networks, including the default mode network (DMN). We also found atypical associations between DMN activation and ADHD subjects’ inattentive symptoms and task performance.

**Conclusion:** The current study demonstrated rightward VNA in ADHD during a simple letter discrimination task. This result adds an important novel consideration to the growing literature identifying abnormal visual processing in ADHD. We postulate that this characteristic reflects greater perceptual engagement of task-extraneous content, and that it may be a basic feature of less efficient top-down task-directed control over visual processing. We additionally argue that abnormal DMN function may contribute to this characteristic.

## Introduction

Abundant research has identified abnormal frontal-striatal brain function in attention-deficit hyperactivity disorder (ADHD) (1). However, a growing body of work now also implicates abnormal posterior brain functions and associated abnormalities of early-stage sensory information processing (2). These domains have not yet been conceptually integrated, and we suspect this partly underlies why the more recent findings implicating abnormal sensory processing have been slow to gain widespread interest. The current study seeks to address this issue. First, we examine a specific aspect of low-level information processing in ADHD that our and others’ work have identified to be abnormal. Next, we explore how this characteristic relates to several large-scale distributed network systems, many of which are implicated in ADHD (2, 3). Our goal is to help further substantiate and characterize abnormal information processing in ADHD, and to examine whether and how this characteristic relates to network-level brain functions.

Multiple functional imaging studies have shown abnormal activation or metabolic effects during rest and/or sub-executive operations in ADHD (4–12), which clearly indicates that ADHD abnormal brain function is not limited to higher-order operations. More direct support for low-level sensory information processing deficits comes from several sources. A recent meta-analysis of fMRI studies examining task-based cognition in ADHD identified visual cortical abnormalities to be a key finding in ADHD (2). Abnormal visual cortical structure has also been identified (13). Event-related potential (ERP) studies also directly implicate early sensory processing abnormalities in ADHD (i.e., abnormal N1, N2, P2, and P3) [for review see Ref. (14)], while neurocognitive studies provide additional strong evidence for both perceptual processing (15–18) and naming speed deficits (19–26).

Our research in this domain begins with the precept that complex task-directed actions are likely to rely on a specific manner of sensory information processing that facilitates fast categorical parsing of sensory data. To illustrate, if a person wants to find a pen on a cluttered countertop in order to sign a document, it is task-adaptive to quickly identify (i.e., categorize) that stimulus using the minimal sensory detail required. Here, the pen’s esthetic details and any surrounding content are task-extraneous. Alternatively, if an artist wants to paint a still-life portrait of this pen, they should indulge as much detail as possible. One approach seeks to identify a stimulus using the minimal sensory detail required. The other seeks to indulge as much sensory detail as possible in order to produce a prolonged sensory-immersive experience. We theorize that ADHD involves a reduced capacity for the former mode of processing.

This task-specialized manner of sensory information processing likely depends on the coordinated function of multiple distributed brains systems that get dynamically integrated in service to task-directed actions [for full description of this model see Ref. (27)]. In this view, any impairment to this system, no matter the cause, should result in less efficient task-directed top-down control over sensory information processing, with an associated increased exposure to task-extraneous content. In other words, poor task-directed sensory information processing should result in a greater proportion of off-task visual sensory details being processed. This bias toward sensory immersion/detail over categorical processing may be indexed by an increased contribution of right-lateralized visuo-perceptual processing.

Evidence from our previous behavioral laterality studies in ADHD adults supports the presence of a right-hemisphere bias. These demonstrated greater RH contribution to processing task stimuli, associated left hemisphere (LH) linguistic impairments, and abnormal interhemispheric interaction (28–30). This work also showed that this pattern was reflective of an abnormal brain-state orientation (rather than capacity) (29), bore advantages for RH specialized abilities (29), and impacted high-order cognition (30). Using fMRI and EEG, we further uncovered that RH bias in ADHD was only evident during sub-executive operations (11), exhibited stronger expression with greater ADHD family loading (31), and stronger expression among carriers of the DRD4-7 repeat allele and other ADHD risk-factors. Finally, a robust and literature-consistent (32, 33) biomarker was identified. ADHD subjects exhibit pronounced rightward EEG beta (16–21 Hz) asymmetry in inferior parietal brain regions during Conner’s continuous performance test (CPT) (34), a finding we he have since replicated (35).

Although not yet widely understood, this pattern of findings is well aligned with extant ADHD literature. As noted, slow naming speed is identified in ADHD, which is consistent with impoverished LH contribution to sensory encoding. Previous behavioral laterality studies of ADHD have also indicated increased RH contribution (36, 37). Functional imaging studies at rest or during simple (i.e., sub-executive) challenges have shown a pattern of reduced LH (4–6, 8), and/or increased RH contribution (7, 9–12), and recent diffusion tensor imaging studies have reported greater RH parietal (38) and frontal (39) fractional anisotropy in ADHD. Furthermore, a lack of normally occurring L > R asymmetry in prefrontal cortical convolution complexity has been reported (40), as well as increased RH visual cortex volumes (13). Finally, identified abnormal posterior corpus callosum size (41) and function (7, 42–44), including atypically reversed posterior callosal transfer speeds (44), clearly implicate abnormal integration of verbal and perceptual sensory encoding functions.

A similar pattern of reduced LH and increased RH contributions is evident during more complex tasks; however, this literature is more variable, showing diffuse effects consistent with multiple weaknesses across distributed brain-systems (2, 45–48). Still, several studies have shown greater association between ADHD subjects’ behavioral performance and right-sided brain structure and function (49–56), and EEG studies that have directly examined activation asymmetries and/or that directly compared left–right differences have consistently shown R > L patterns in posterior brain regions (7, 9, 12, 31–34). Finally, a recent meta-analysis of ADHD functional imaging has reported hyper-activation of the strongly right-lateralized ventral-attention network (VAN), noting it may be related to increased distractibility in this population (2), which is consistent with reports showing that greater activation in this network is associated with attentional shifting and/or bottom-up visuo-perceptual processing (57–60).

Thus, the literature strongly implicates some form of increased weighting of non-verbal sensory processing in ADHD. We hypothesize that this stems from variable impairments to task-directed brain functions that otherwise facilitate fast/efficient identification and verbal encoding of task relevant stimuli (for model description: (27)). Still, abnormal processing asymmetry has been inconsistently observed during complex EF-level operations. We suspect that this is because the operative feature of abnormal sensory information processing in ADHD is the relative, rather than absolute, contribution of left- and right-hemisphere sensory functions, and few studies are designed to identify such effects.

Methods for the direct analysis of EEG asymmetry are well developed, and as noted, have consistently shown R > L patterns in ADHD. However, related fMRI methods to assess the asymmetry of BOLD signal have only recently begun to overcome methodological difficulties involving thresholding techniques (61, 62). The current study utilizes these novel fMRI methods to further examine and substantiate abnormal information processing asymmetry in ADHD. Our previous studies indicated that ADHD rightward biased processing is maximally evident during linguistic challenges, and that it underlies linguistic impairments (28–30, 34). Hence, we utilized an fMRI paradigm that presents word stimuli and requires subjects to make either a letter discrimination or spatial judgment in different blocked conditions. This task has been previously shown to elicit lateralized activations for the letter and spatial judgments (63). Given the exclusive use of word stimuli in this study, we hypothesized that ADHD children would show a general pattern of increased rightward asymmetry in visual cortical regions compared to controls. However, we also hypothesized that this effect would be maximally robust during the letter discrimination condition that requires a more fixed attentional set and fast verbal categorizations.

Furthermore, since we theorize that asymmetry in low-level perceptual processing is directly related to abnormalities in higher-level processing, we sought to understand the relationship between perceptual asymmetry and activity in other brain networks. Patterns of intrinsic functional connectivity in the brain have revealed multiple networks of brain regions whose activity is correlated during rest (64). Several of these networks have been implicated in ADHD (2, 3). Among these, abnormal default mode network (DMN) function has been the most widely reported (2). Although previously understood as a resting or task-negative network (65), the DMN is now also understood to play a role in internally directed self-referential aspects of cognition (66), including internal aspects of task-directed cognition (67, 68). In fact, recent work has even suggested a link between DMN integrity and verbal working memory capacity (69). This raises the intriguing possibility that abnormal DMN function in ADHD might be associated with a reduced capacity to orchestrate the internal aspects of task-directed cognition (e.g., planning, sequencing, maintaining, and updating task directives) (70–73), possibly undermining a general capacity for task-directed brain functions, including task-specialized sensory information processing (74). To examine this possibility, the current study examines, as a secondary aim, whether visual processing asymmetry in ADHD is uniquely associated with DMN function. To test the specificity of any such effects other networks are also examined.

## Materials and Methods

### Subjects

Subjects were recruited from Los Angeles County and the surrounding regions using a database of participants from previous UCLA studies who indicated they were willing to participate in future studies. Subjects were also recruited through flyers posted near UCLA, and advertisements on focus group websites (e.g., parenting blogs). Given our interest to examine ADHD-specific asymmetry effects, we chose to limit possible variability in brain-laterality due to gender, handedness, and/or variation in pubertal onset (75). Accordingly, participation required being male, right-handed, and between the ages of 11 and 17, with an initial parental report that the child had begun using deodorant, with puberty-onset later confirmed by a parent during a private interview.

After receiving verbal and written explanations of study requirements a parent and the participating child provided written informed consent/assent, as approved by the UCLA Institutional Review Board. To screen for ADHD and other psychiatric disorders using DSM-IV criteria, participating children and their mothers were interviewed using the semi-structured interview of the Schedule for Affective Disorder and Schizophrenia for School-Age Children-Present and Lifetime Version (K-SADS-PL) (76). Autism was ruled out via the social communication questionnaire (77). Diagnostic interviews were conducted by a highly trained clinical interviewer (MA Psychology), after which, “best estimate” diagnoses were determined from individual review of diagnoses, symptoms, and impairment level by a board certified child psychiatrist (78). Inclusion of ADHD subjects required a current diagnosis of ADHD (six or more symptoms on inattentive and/or hyperactive subscales). Inclusion of non-ADHD controls required no evidence of past or current ADHD (i.e., reporting four or fewer ADHD symptoms on inattentive and hyperactive subscales), and no known cases of ADHD among first degree relatives. Subjects were excluded based on the following criteria: past or current documented neurological disorder, a significant head injury resulting in loss of consciousness, a diagnosis of schizophrenia or autism (self or first degree relative), or an estimated full scale IQ < 80.

Handedness was assessed with a shortened version of the Edinburgh Handedness Inventory (79). This scale uses seven questions regarding hand preference and produces scores ranging from negative 14 (indicating maximum left-handedness) to positive 14 (indicating maximum right-handedness). Assessment of verbal ability was performed to help rule out the possibility of undiagnosed comorbid reading difficulties in ADHD contributing to asymmetry effects. We used age normed scores from the vocabulary subtest of the Wechsler’s intelligence test for Children third edition (WISC-III) (80), the reading and spelling subtests of the Peabody Individual Achievement Test-Revised (PIAT-R) (81), and the word-attack (phonemic awareness) subtest of the Woodcock–Johnson-Revised (WJ-R) (82). Subject demographic information is presented in Table 1. All subjects were enrolled in age-appropriate educational programs as required by California law. Subjects on stimulant medication were asked to discontinue use for 24 h prior to their visit.

**Table 1:** **Sample demographics**.

Clinical variables	Controls *N* = 21	ADHD *N* = 21	Statistic
IQ	$\bar{x}=112.7$, STD = 18.2	$\bar{x}=110.6$ STD = 15.6	*t* = 0.39, *p* = 0.70
Age	$\bar{x}=13.1$, STD = 1.5	$\bar{x}=13$, STD = 1.6	*t* = 0.29, *p* = 0.77
SES	$\bar{x}=2.9$, STD = 0.85	$\bar{x}=2.2$, STD = 0.87	*t* = −2.5, *p* = 0.02
ADHD type	N/a	11C, 10I	N/a
Anxiety	0 affected	1 affected (GAD)	N/a
Mood	0 affected	0 affected	N/a
ODD	0 affected	6 affected	fe: *p* = 0.02
CD	0 affected	1 affected	N/a
Handedness score	$\bar{x}=12.7$, STD = 2.8	$\bar{x}=12.1$, STD = 2.7	*t* = 0.75, *p* = 0.46
Vocabulary	$\bar{x}=12.5$, STD = 3.5	$\bar{x}=11.9$, STD = 3.6	*t* = 0.54, *p* = 0.59
Phonology	$\bar{x}=106.5$, STD = 12.5	$\bar{x}=103.5$, STD = 12.4	*t* = 0.44, *p* = 0.44
Reading	$\bar{x}=110$, STD = 9.6	$\bar{x}=102.4$, STD = 14.2	*t* = 1.98, *p* = 0.06
Spelling	$\bar{x}=108.9$, STD = 14.4	$\bar{x}=98.3$, STD = 13.5	*t* = 2.4, *p* = 0.02

*IQ, estimated from block-design and vocabulary subtest of WISC-III; SES, socioeconomic status measured by Hollingshead (83) scale; ADHD type: C, combined; I, inattentive; handedness score = 14 point scale from Edinburgh Handedness Inventory, with 14 indicating maximum right-handedness (see text for description and reference); Anxiety/mood reflects definite diagnosis of at least one current anxiety and/or mood disorder as assessed by direct interview using K-SADS-PL; ODD/CD, oppositional defiant disorder and conduct disorder as assessed by direct interview using the K-SADS-PL. fe, Fisher’s exact test; see text for description of linguistic measures*.

Thirty-one ADHD and 25 typically developing right-handed male children between the ages of 11 and 16 underwent fMRI procedures. Ten ADHD subjects were excluded (5 = motion, 2 = non-compliance, 1 = sleep, 1 = non-tolerant of fMRI environment, and 1 = image distortion from permanent retainer). Four control subjects were excluded (1 = medical problem that impacted brain development, 1 = father diagnosed with ADHD, 1 = borderline ADHD, and 1 = non-tolerant of fMRI environment). The final sample consisted of 21 ADHD and 21 control subjects. The ADHD sample was 81% Caucasian, 14% African American, and 5% Hispanic. The control sample was 62% Caucasian, 9.5% African American, 19% Hispanic, and 9.5% Asian.

### Task procedures

The fMRI task was adapted from a previous block-design study that uncovered robust laterality differences for “letter” versus “spatial” processing in healthy adults (63). In this study, subjects viewed successive presentations of four-letter words presented in black-font with a red letter in the second or third position and had to decide whether the red letter was on the left or right (location condition) or an “A” or not (letter condition). Subjects responded via button presses, using the index finger to signal a “left” or “yes-A” response, and the middle finger to signal a “right” or “not-A” response. During baseline, subjects responded when a word appeared (i.e., no decision). Thus, the stimuli in all three conditions (letter, location, and baseline) were identical, and only the task-instructions differed. The original study used lateralized presentations, however, the authors did not report brain activation differences based on visual field, and so we used central presentations. We also did this to reduce complexity and difficulty given our use of an impaired child sample. The original study also alternated response hand within subjects. We used right-handed responses to assure response related brain activation was eliminated against baseline, and again to avoid unnecessary complexity while working with a child ADHD sample. The original study also used German words. We used English words.

Stimuli were generated using the MRC Psycholinguistic Database (84). They consisted of 192 four-letter concrete nouns assessed for word-frequency (Kucera–Francis and Thorndike–Lorge), concreteness, and imagability, and matched across key stimulus parameters (left versus right, “A” versus “Not-A”). Half of the words contained a target letter “A,” and half did not. For each of these sets, the target occurred an equal number of times in the second or third position (i.e., left or right). Baseline conditions used four additional unique stimuli (FLAP, HAND, MILK, and CORD).

Two data collection runs were performed. Each presented eight task-blocks (four location and four letter) interspersed with seven baseline conditions. Within runs, the order of task-blocks was randomized, with pre-block instruction screens indicating which task to perform. Task-blocks contained 12 2-s randomly jittered trials (±250 ms) – 6 with target “A”s, 6 without, and among these sets, an equal number of targets in the second or third position. The order of trial types was randomized within blocks. During trials, words were presented centrally for 150 ms in all capital 48-point black-font (except for the red target letter). A central fixation cross was displayed between stimulus presentations. Baseline conditions contained eight trials and used the same stimulus presentation parameters. The 192 task-stimuli were newly randomized for each subject, with no stimuli repeating across both runs. Stimulus presentation and response collection were controlled using MATLAB (The Mathworks, Inc.) and the Psychophysics Toolbox (85). See Part 1 in Supplementary Material for graphical portrayal of task parameters.

### General procedures

fMRI procedures were a component of a broader protocol. On the first day, subjects underwent clinical, cognitive, and EEG assessments. On the second day, they underwent fMRI consenting, safety screening, training, and testing. The mean time difference between days 1 and 2 was 91.5 days for ADHD subjects, and 56.3 days for controls (no statistical group difference). Before fMRI scanning, task training occurred via a standardized computer program implemented using E-prime software (Psychology Software Tools, Inc.). Although the program was designed to operate automatically, research staff read aloud the instructions and prompted subjects to repeat any training module not performed above chance. Task training was performed to reduce the likelihood of capturing brain activation associated with task learning during scanning procedures.

The program first introduced subjects to each of the task conditions. This required active participation as subjects learned about stimuli and associated response mappings for each condition (location, letter, and baseline). Each of these modules ended with a practice that provided trial-by-trial and overall performance feedback. Next, the program portrayed the intermixing of blocked-conditions and associated instruction screens that signaled which task to perform. The instruction screens were identical to those used in the scanner. These screens were designed to signal which task to perform next, and provide prompts to help children remember condition-specific response mappings (i.e., instruction screen graphics displayed associated response mappings). This task-mixing practice section also ended with a brief practice that provided overall performance feedback. Finally, subjects underwent a mock run of the experiment exactly as presented in the scanner, barring a few differences (different word stimuli, keyboard responses, and overall performance feedback).

Task training took approximately 30 min, after which, subjects and a parent walked to the fMRI facility, where they waited in a lounge during set-up. During this time, subjects were encouraged to explore a nearby mock-scanner, listen to recordings of MRI and fMRI scanner noises, and practice inserting earplugs. After fMRI equipment and software set-up was complete, subjects entered the scanner control room and were given an opportunity to become familiar/comfortable with the environment, as well as select a movie to watch during set-up and structural imaging. Upon entering the scanner-room, subjects were instructed to use the critical equipment (response box, head phones, goggles, and emergency button) and were allowed to watch their selected movie (via fMRI goggles) during additional set-up and shimming procedures. Throughout these and subsequent scanning procedures, a concerted effort was made to keep children actively engaged and comfortable.

Before running the fMRI task, children were shown a “start screen.” This reminded them what each of the instruction screens looked like and repeated general task-instructions. A research staff read the instructions to the subject and prompted them to demonstrate button presses associated with each condition. This assured us that children were using the button box correctly, and it made the subjects aware that we were able to monitor their button presses in real-time.

### Data acquisition

This study was conducted at the Staglin IMHRO Center at UCLA. MRI recording was performed with a standard 12-channel head coil on a Siemens 3T Trio Magnetic Resonance Imaging System with TIM. Two functional runs including 195 volumes each were acquired. These images were collected over 33 axial slices covering the whole cerebral volume using a T2*-weighted gradient-echo sequence (TR = 2000 ms, TE = 30 ms, flip angle = 78°, matrix size 64 × 64, 3-mm in-plane resolution, 3-mm thick slices, and 0.75-mm gap). For each participant, a high-resolution MP-RAGE structural volume was also acquired (TR = 1900, TE = 2.26, and flip angle = 9°) with 176 sagittal slices, each 1 mm thick with 1 mm × 1 mm in-plane resolution.

### fMRI data analysis

Analysis was carried out using FSL’s FMRI Expert Analysis Tool (FEAT) Version 5.1 (FMRIB’s Software Library, www.fmrib.ox.ac.uk/fsl). Data preprocessing involved the following steps: motion correction (86), brain extraction (87), slice timing correction, spatial smoothing with a 10-mm FWHM Gaussian kernel, high pass temporal filtering using Gaussian-weighted least-squares straight line fitting with sigma = 90.0 s, and pre-whitening (88). For each run, the BOLD response was modeled using a separate explanatory variable (EV) for each task condition (letter and location). For each task condition, the presentation design was convolved with a gamma function to produce an expected BOLD response. The temporal derivative of this time-course was then included in the model for each EV to capture any unexpected temporal shifting, and motion correction parameters were also included in the design as additional nuisance regressors. Data for each condition were then fitted to the model using FSL’s implementation of the general linear model.

Each subject’s statistical data were then warped into a standard-space based on the MNI-152 atlas. We used FLIRT to register the functional data to the atlas space in three stages. First, functional images were aligned with the high-resolution co-planar T2-weighted image using a six-degrees-of-freedom rigid-body warping procedure (86, 89). Next, the co-planar volume was registered to the T1-weighted MP-RAGE using a six-degrees-of-freedom rigid-body warp. Finally, the MP-RAGE was registered to the standard MNI atlas with a 12-degrees-of-freedom affine transformation, and then this transformation was refined using FNIRT non-linear registration (90, 91).

After analyzing each functional run for each subject, the two functional runs were combined using a fixed-effects analysis. Data from each subject were then passed into a higher-level analysis, which allowed comparisons within and between groups. Higher-level analysis was carried out using FLAME (FMRIB’s Local Analysis of Mixed Effects), such that group-level effects were modeled using random effects (92). Z (Gaussianised T/F) statistic images were thresholded using clusters determined by *Z* > 2.3 and a cluster significance threshold of *p* < 0.05 (corrected) (93, 94). To examine individual differences, additional higher-level analyses were performed using behavioral (task accuracy and reaction time) and psychological assessment measures (ADHD symptom measures) as cross-subject regressors. These analyses were performed in FEAT, using a FLAME higher-level analysis that modeled the mean across subjects with one EV, and the demeaned behavioral correlate with a second EV. This resulted in whole brain maps for each regressor that reflected the degree to which each voxel’s activity correlated with that regressor across subjects. Positive and negative contrast maps were thresholded according to the same *Z* > 2.3, cluster size *p* < 0.05 threshold.

### Asymmetry analysis

**Figure 1:**
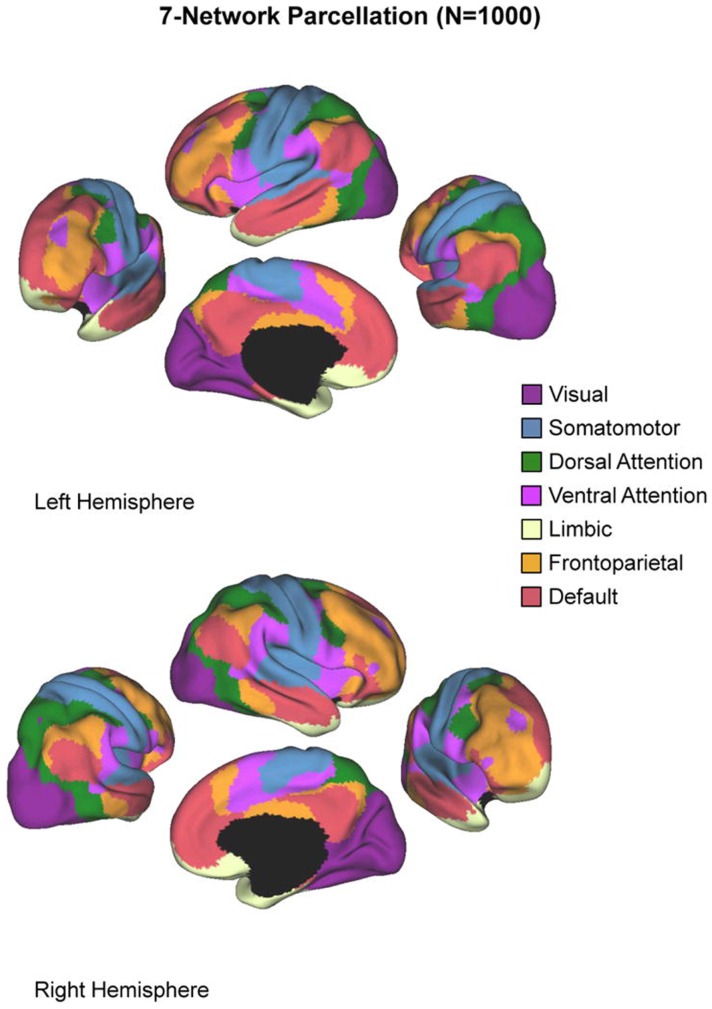
**The seven-network parcelation**. Figure reproduced from Castellanos and Proal (3).

The purpose of our asymmetry analysis was twofold. First, we intended to characterize brain asymmetry in patients and controls in visual processing regions of the brain, i.e., those regions involved with the perceptual processing of the stimuli during the task. Second, we intended to probe how asymmetry in visual areas was related to processing in several key networks throughout the brain, several of which are suspected to play a role in ADHD. Recent work in neuroimaging has shown that the brain can be parceled into distinct networks based on intrinsic functional connectivity at rest, and that these networks may represent meaningful cognitive units (95–97). Several of these networks show altered activity in individuals with ADHD (2, 3). Here, we follow Castellanos and Proal (3) in employing the seven-network parcelation derived by Yeo et al. (64). This network parcelation comes from analysis of resting-state fMRI from 1000 healthy adult subjects. Yeo et al. used a clustering algorithm to divide the brain into seven non-overlapping networks on the basis of functional coupling, yielding a series of masks registered to the standard MNI-152 space that we used in our analysis. The seven networks are depicted in Figure 1, and are known by their associations with the neuroimaging literature as the visual, somatomotor, dorsal attention, ventral-attention, limbic, frontoparietal, and default networks. We computed the asymmetry index (AI) using voxels only within the visual network, and then correlated the AI with activation measures derived from each of the other networks. Note that these networks are non-overlapping, so voxels contributing to the visual network asymmetry are not included as part of any other network.

The most common approach to quantifying asymmetries of functional brain activation in the neuroimaging literature is to compute an AI as the ratio of the difference between left hemisphere activation (LHA) and right-hemisphere activation (RHA) and the sum of activation in both hemispheres:

AI=(LHA−RHA)∕(LHA+RHA)

Positive AI values up to a maximum of +1 correspond to greater LH lateralization, while negative values with a minimum of −1 correspond to right-hemisphere lateralization. However, there is little consensus as to how to compute the activation values that enter into this equation. Typically, voxels above a specified threshold are counted (98–102) or their statistical values are summed or averaged (103–105). Importantly, the choice of statistical threshold can have an effect on computed AI values (61, 98, 106–109).

Two types of strategy for dealing with thresholding issues have recently emerged: (1) AI values are computed across a range of threshold values instead of a single threshold, and laterality curves are presented (62, 106, 107, 110, 111), or (2) a single AI value is computed for each subject using the distribution of AI values across thresholds either to select a reasonable threshold or to combine across thresholds using a weighting function (108, 112–115). We have chosen to use a combination of both strategies. First, we computed a single AI value for each subject using a variation of Wilke and Lidzba (61) “adaptive threshold” technique. Here, for each subject, mean voxel intensity within the visual network mask was used as the threshold to compute a visual network asymmetry index (VN-AI). Next, in order to thoroughly characterize activation asymmetries in each group we present asymmetry curves across a range of statistical thresholds.

Asymmetry indexes were computed using the iBrain Laterality Toolbox (62), using standard-space *z*-score images for each subject for each contrast. *Z*-score images were masked by the visual network mask from Yeo et al. (64), and split into left and right halves along the midline of the brain. Then, to generate “adaptive threshold” AIs, individual’s images were thresholded according to their mean voxel intensity within the visual network, and averages were computed within the left and right halves of the mask and subjected to the AI calculation described above. To generate AI-curves, the same approach was utilized except that images were thresholded multiple times in 0.1 increments from *z* = 0.1 to *z* = 3.1 (corresponding to the *z*-distribution *p*-value of 0.001) creating 31 different AI scores.

Group differences in the adaptive-threshold based VN-AI were examined for each condition (all–baseline, letter–baseline, and location–baseline) using univariate ANOVA (adjusted for age), and are considered our primary analyses of visual network asymmetry. AI-curves are included mainly for visual inspection; however, a “principal components analysis” (PCA) based assessment of AI-curves is also presented as a secondary statistical approach.

Note that contrasts used in these asymmetry analysis (letter–baseline, location–baseline, and all–baseline) involved comparison of conditions that had identical visual stimuli, and thus produced modest visual network activation. The primary adaptive-threshold approach contends with this by normalizing each subject’s AI score to their own mean signal strength within the visual network. However, with AI-curves, the maximum *z*-value shared by all subjects was *z* = 2.0. Thus, PCA based analysis of AI-curves targets *z*-values up to that point (i.e., that includes our full sample). We additionally report PCA based assessment of AI-curves up to *z* = 3.1, noting the reduction in sample sizes [sample sizes at *z* = 3.1: all–baseline (18 controls, 17 ADHD), letter–baseline (17 control, 16 ADHD), and location–baseline (15 controls, 16 ADHD)].

Group differences across the 20 *z*-thresholds comprising the portion of the AI-curves that contained our full sample (from *z* = 0.1 to *z* = 2.0) were assessed using principal component analysis (PCA). Here, in order to reduce the number of dimensions from these 20 AI values (at each *z*-threshold) PCA was conducted on standardized variables (correlations) and components with eigenvalues >1 extracted. Because the primary focus was on a single component explaining the variability in measures computed using different thresholds, no rotation was utilized. The same approach was used to assess group differences in AI-curve values ranging from *z* = 0.1 to *z* = 3.1 as a sensitivity analysis. In both cases, group differences in the resultant components were assessed using univariate ANOVA (adjusting for age).

Finally, a key goal of the current study was to examine the relationship between hypothesized visual processing asymmetries in ADHD and identified functional networks suspected to play a role in the disorder (2, 3, 64). To do this, we computed the average *z*-score within each network mask for each subject (representing task-related activity within that network), and correlated these values (adjusted for age) with the adaptive-threshold based VN-AI values.

### Behavioral analyses

Group differences in letter- and location-task behavioral performance (accuracy and response time) were tested using univariate ANOVA (adjusted for age). Two additional analyses used partial correlations (adjusted for age) to examine the relationship between letter-task performance and VN-AI values, and mean signal intensity across the six extra-visual networks examined in this study.

### Correlation analyses

Where relevant, we used Fisher’s *r*-to-*z* test to statistically examine the difference between two correlations (116). First, the correlations are transformed so that they are unbounded, using the inverse hyperbolic tangent function. Next, the difference between the transformed correlations is converted to a *Z*-score based on the sample sizes, and then a *p*-value is obtained based on the *Z*-score.

## Results

### Behavior

#### Task performance

Controls exhibited better accuracy during the letter task, and a trend suggested the same during the location task (Table 2). Partial correlations (adjusted for age) indicated a speed–accuracy tradeoff during the letter task among ADHD subjects (accuracy correlated with response time: *r* = 0.74, *p* < 0.000), with a trend showing the same pattern during the location task (*r* = 0.39, *p* = 0.09). Fisher *r*-to-*z* test indicated that the ADHD speed–accuracy tradeoff during the letter task was significantly different from controls (*z* = 2, *p* = 0.04).

**Table 2:** **Group differences in behavior**.

Behavior measure	Controls		ADHD		Statistic		
	$\bar{x}$	SE	$\bar{x}$	SE	*f*	df	*p*
Letter Accuracy	0.91	0.018	0.86	0.018	4.4	2, 39	0.043
Letter RT	590	21	590	21	0.007	2, 39	0.93
Location Accuracy	0.93	0.017	0.88	0.017	3.29	2, 39	0.08
Location RT	490	17	500	17	0.141	2, 39	0.71

*Univariate analysis of variance (adjusted for age) was used to examine group differences in tasks accuracy and response time (RT); $\bar{x}$ = estimated marginal means; SE = standard error; Accuracy = proportion correct; RT values in milliseconds*.

### Standard neuroimaging results

#### Tasks–baseline

##### All–baseline

**Figure 2:**
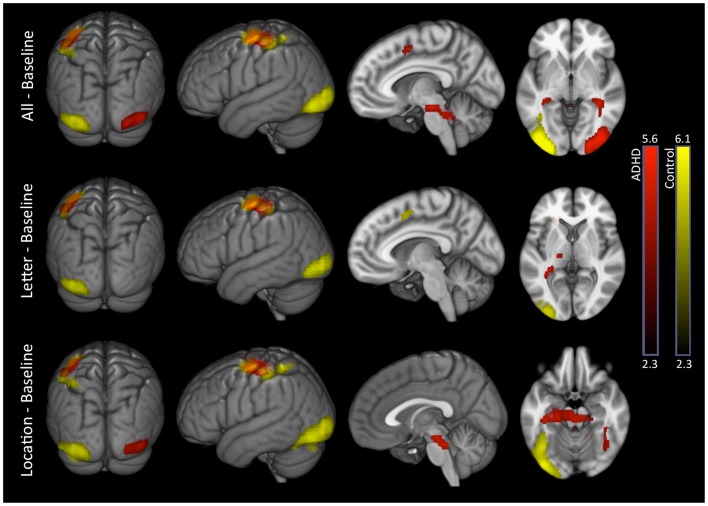
**Task conditions–baseline: within-group effects**. Within-group analysis of BOLD signal revealed unique LH occipital activations in controls (yellow) and unique RH occipital activations in ADHD subjects (red). Several common activations were also evident (orange), such as LH, supplementary motor, pre-central gyrus (superior lateral, inferior medial), and post-central gyrus boarding the supramarginal gyrus. ADHD subjects showed additional unique subcortical activations that included thalamus, brainstem, cerebellum, and hippocampus. Images are thresholded using a voxelwise threshold of *z* = 2.3 and a cluster size probability of *p* < 0.05.

Both groups exhibited significant activation of the occipital cortex (extending into fusiform regions), but in opposite hemispheres (RH in ADHD, LH in controls). ADHD and controls also exhibited several overlapping activations in LH brain regions that included supplementary motor, pre-central gyrus (superior lateral, inferior medial, plus inferior lateral in controls), and post-central gyrus bordering the supramarginal gyrus (extending into superior parietal cortex in controls). Lastly, ADHD subjects showed additional unique activations in the brain stem and cerebellum (Table 3; Figure 2).

**Table 3:** **Condition–baseline: within-group effects**.

			Control	ADHD
	
Region	Hem.	MNI	*Z*-val.	*Z*-val.
**ALL–BASELINE**				
Supplementary motor cortex	L	−10, 2, 50	2.93	3.4
Superior lateral pre-central gyrus	L	−40, −12, 66	6.03	5.63
Inferior lateral pre-central gyrus	L	−44, −2, 28	4.05	None
Inferior medial pre-central gyrus	L	−24, −12, 50	5.02	4.34
Supramarginal gyrus	L	−38, −36, 38	4.83	3.93
Occipital cortex	L	−36, −94, −4	5.62	None
Occipital cortex	R	40, −82, −4	None	4.29
Brain stem	Mid	−4, −22, −16	None	3.72
Brain stem	Mid	0, −38, −24	None	4.07
Cerebellum lobule VI	R	26, −46, −28	None	5.04
**LETTER–BASELINE**				
Supplementary motor cortex	L	−10, 2, 50	2.37	None
Superior lateral pre-central gyrus	L	−40, −12, 66	5.24	4.87
Inferior lateral pre-central gyrus	L	−44, −2, 28	3.80	None
Inferior medial pre-central gyrus	L	−24, −12, 50	4.20	3.90
Supramarginal gyrus	L	−38, −36, 38	4.44	3.93
Occipital cortex	L	−36, −94, −4	5.17	None
Thalamus	L	−18, 20, 6	None	2.93
**LOCATION–BASELINE**				
Supplementary motor cortex	L	−12, 2, 46	2.53	None
Superior lateral pre-central gyrus	L	−40, −12, 66	5.16	4.90
Inferior lateral pre-central gyrus	L	−46, −2, 26	3.60	None
Inferior medial pre-central gyrus	L	−24, −12, 50	5.14	4.34
Supramarginal gyrus	L	−36, −36, 36	4.50	3.66
Occipital cortex	L	−32, −92, −4	4.98	None
Occipital cortex	R	34, −86, −4	None	4.0
Brain stem	Mid	2, −26, −18	None	3.31
Brain stem	Mid	0, −34, −24	None	3.47
Cerebellum lobule VI	R	24, −48, −28	None	3.91
Hippocampus	L	−32, −26, −14	None	3.53
Hippocampus	R	32, −34, −4	None	2.60

*Table shows significant within-group activations per condition ordered along anterior-to-posterior axis. Hem., hemisphere; L, left hemisphere; R, right hemisphere; Mid, activated voxel with *x*-coordinate between −5 and 5; MNI, Montreal Neurological Institute structural atlas coordinates (*x*-, *y*-, and *z*-axis); *Z*-val., *z*-value indicating BOLD signal intensity at reported voxel; Significance determined using a voxelwise threshold of *z* = 2.3 and a cluster size probability of *p* < 0.05*.

##### Letter–baseline

This contrast showed the same basic pattern as all–baseline except for the following: only controls activated supplementary motor cortex; significant occipital, brain stem, or cerebellum activations were not present in ADHD subjects; and ADHD subjects showed a unique activation in the left thalamus (Table 3; Figure 2).

##### Location–baseline

This contrast showed the same basic pattern as all–baseline, as well as additional unique hippocampal activations among ADHD subjects (Table 3; Figure 2).

Direct comparison between groups did not show significant differences.

#### Task comparisons

##### Letter–location

There were no significant effects for this contrast.

##### Location–letter

**Figure 3:**
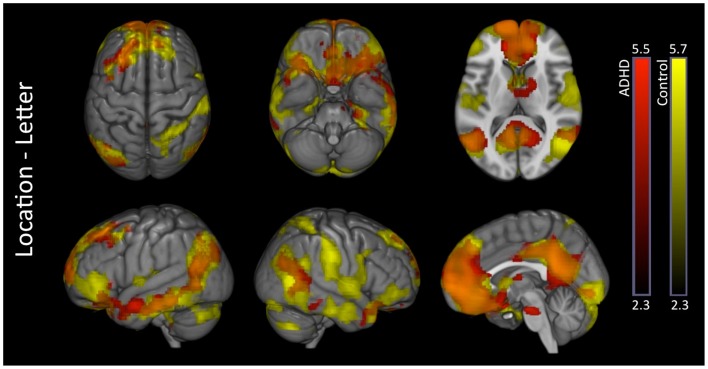
**Location–letter: within-group effects**. Within-group analysis of location–letter contrast revealed several overlapping activations among ADHD (red) and control subjects (yellow) likely reflective of greater DMN activation during the location versus letter tasks. ADHD subjects showed additional unique activations in subcortical structures, while controls showed additional unique activations within somatomotor network regions. Overlap between the two groups is shown in orange. Images are thresholded using a voxelwise threshold of *z* = 2.3 and a cluster size probability of *p* < 0.05.

Attention-deficit hyperactivity disorder and control subjects exhibited several overlapping activations in brain regions associated with the DMN (medial prefrontal, medial parietal, and inferior parietal cortices). Additional unique activations were evident in subcortical regions among ADHD subjects, and within somatomotor regions among in controls (Figure 3 – see Part 2 in Supplementary Material for details). Direct comparison between the groups did not show significant differences.

### Asymmetry analyses

#### Adaptive-threshold based asymmetry

**Figure 4:**
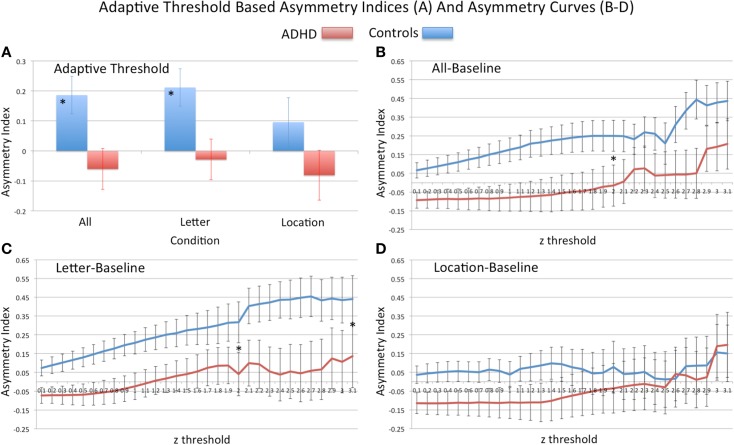
**Analysis of visual network asymmetry**. Showing adaptive-threshold based visual network asymmetry indices (AN-VI) **(A)** and asymmetry curves, AI-curves **(B–D)**. For adaptive-threshold indices “*” signifies group difference in all–baseline and letter–baseline conditions. For AI-curves **(B–D)** “*” signifies group difference in primary PCA component computed from *z*-thresholds ranging from 0.1 to 2.0 (all–baseline and letter–baseline), and to 3.1 (letter–baseline). At the threshold *z* = 3.1, *n* sizes reduced to the following: all–baseline (18 controls, 17 ADHD), letter–baseline (17 control, 16 ADHD), and location–baseline (15 controls, 16 ADHD); Note: positive asymmetry values = leftward asymmetry.

Analysis of group differences in the adaptive-threshold based VN-AI showed that controls had significantly greater leftward asymmetry for the all–baseline and letter–baseline contrasts (Table 4; Figure 4A).

**Table 4:** **Group difference in adaptive-threshold based visual network asymmetry**.

fMRI contrasts	Controls		ADHD		Statistic		
	$\bar{x}$	SE	$\bar{x}$	SE	*f*	df	*p*
All–baseline	0.18	0.06	−0.06	0.06	6.8	1,39	0.013
Letter–baseline	0.21	0.06	−0.03	0.06	6.5	1,39	0.014
Location–baseline	0.09	0.08	−0.08	0.08	2.2	1,39	0.15

*Univariate analysis of variance (adjusted for age) was used to examine group differences in the adaptive-threshold based visual network asymmetry indices (VN-AIs); $\bar{x}$ = estimated marginal means; SE, standard error*.

Although ADHD subjects did not differ on vocabulary and phonological measures, a trend effect (*p* = 0.06) suggested poorer reading, while a significant effect (*p* = 0.02) indicated poorer spelling compared to controls (Table 1). Importantly, the ADHD group mean for these standardized measures was not suggestive of clinical impairment (age normed standard mean for these measures = 100, ADHD reading = 102.4; ADHD spelling = 98.3). However, three ADHD subjects had reading and spelling scores within impairment ranges (i.e., 1.5–2 standard deviations below the standardized mean) – one of these learned English as second language. Asymmetry scores from these subjects did not present as outliers, and group differences in VN-AI effects remained significant with these subjects removed, or after co-varying for reading and spelling abilities.

#### AI-curves based asymmetry

##### Principal components analysis of AI-curves

For AI-curve values that contained the full sample (*z* = 0.1–2.0), a single principal component explained most of the variance in AI measures (≥ 91% for all conditions). For the all–baseline condition, only one component had an eigenvalue >1; the remaining conditions each had a second component with an eigenvalue >1, but accounting for a small amount of total variance (<8%). Because the first component for all conditions explained the vast majority of variance in measures and due to our focus on identifying a single measure of asymmetry, only the first component was used in subsequent analyses. A similar pattern of results emerged when conducting the PCA with thresholds up to *z* = 3.1.

##### Group differences in AI-curves

Consistent with primary VN-AI analysis, analysis of PCA asymmetry components derived from thresholds *z* = 0.1–2.0 (i.e., the upper limit that included all subjects) showed controls had significantly greater leftward asymmetry than ADHD subjects during all–baseline and letter–baseline conditions (Table 5; Figures 4B,C). In the letter–baseline condition, this effect remained significant across the full range of threshold values (i.e., up to *z* = 3.1) [*F*(1, 39) = 4.5, *p* = 0.04], even with the associated loss of statistical power (ADHD sample reduced, 24%; control sample reduced, 19%).

**Table 5:** **Group differences in AI-curves**.

fMRI contrasts	Controls		ADHD		Statistic		
	$\bar{x}$	SE	$\bar{x}$	SE	*f*	df	*p*
All–baseline	0.36	0.20	−0.36	0.20	6.3	1,39	0.016
Letter–baseline	0.34	0.20	−0.34	0.20	5.5	1,39	0.024
Location–baseline	0.23	0.22	−0.22	0.22	2.1	1,39	0.16

*Univariate ANOVA (adjusted for age) was used to examine group differences in PCA components derived from asymmetry indices computed for each condition at thresholds ranging from *z* = 0.1–2 (see Materials and Methods section); $\bar{x}$ = estimated marginal means; SE, standard error*.

Please note that additional analyses involving visual network asymmetry exclusively utilized the letter–baseline adaptive-threshold VN-AI metric. Moreover, in the assessment of VN-AI association with extra-visual networks, extra-visual-network values were derived exclusively from the letter–baseline condition where group differences in visual network asymmetry occurred. Also, note from Figure 2 that at a group level the only significant increases in the cortex during tasks relative to baseline are within the visual network itself, and also in the left sensorimotor cortex. We attribute this to the relative similarity between our tasks and the active baseline. Hence, for correlation analyses involving extra-visual network BOLD signal, correlation effects largely reflect associations with variable degrees of task-associated deactivation.

#### VN-AI association with averaged signal in extra-visual networks

Partial correlation analysis (adjusted for age) showed that VN-AI in ADHD subjects was generally and positively correlated with signal in extra-visual networks during the letter task, with the effect surviving Bonferroni correction for the somatomotor, ventral-attention, and DMN. In contrast, controls showed a pattern of negative (but mostly non-significant) associations between VN-AI and signal in extra-visual networks. One effect in controls (i.e., VN-AI correlation to LIM) was significant and survived Bonferroni correction. Fisher’s *r*-to-*z* test indicated that all correlation effects were significantly different between groups (*z*-values ranged between 2.3 and 3.5, *p*-values ranged between 0.02 and 0.0005) (Table 6).

**Table 6:** **Partial correlations (adjusted for age) between VN-AI and averaged signal across functional networks during the letter task**.

Asymmetry		SOM*	DAN*	VAN*	LIM*	FPN*	DMN*
A: VN-AI	*r* *p*	0.66 0.001	0.49 0.03	0.66 0.001	0.40 0.08	0.52 0.02	0.57 0.008
C: VN-AI	*r* *p*	−0.29 0.21	−0.22 0.34	−0.19 0.43	−0.63 0.003	−0.27 0.26	−0.31 0.18

Partial correlations (adjusted for age) were used to examine the association between averaged signal within target networks and VN-AI in each group; A = ADHD; C = Controls; r-values are shown in the top of each cell; p-values are shown in the bottom of each cell; VN-AI, letter-baseline adaptive threshold visual network asymmetry index; SOM, somatomotor; DAN, dorsal attention network; VAN, ventral attention network; LIM, limbic network; FPN, frontoparietal network; DMN, default mode network; *Fisher’s r-to-z test indicate significant group difference; Bold border = effect significant after multiple comparison adjustment (Bonferroni).

#### VN-AI association with voxelwise signal maps

**Figure 5:**
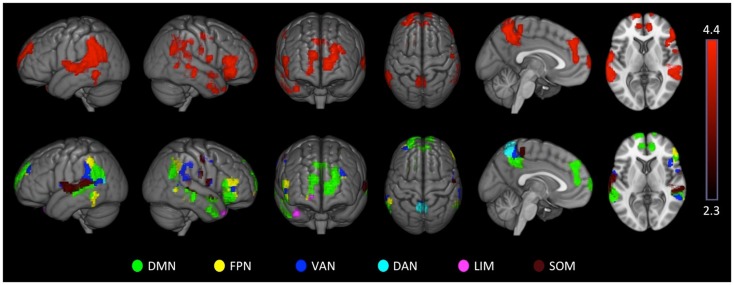
**Attention-deficit hyperactivity disorder visual network asymmetry association with BOLD signal during the letter task**. ADHD subjects showed exclusive positive associations between visual network asymmetry (VN-AI) during the letter task and BOLD response across multiple brain regions, likely reflective of DMN activation. These ADHD-exclusive associations produced significant group differences. The upper row shows ADHD positive association maps. The lower row shows the same maps color-coded to depict extra-visual networks; SOM = somatomotor; DAN = dorsal attention network; VAN = ventral-attention network; LIM = limbic network; FPN = frontoparietal network; DMN = default mode network. Images are thresholded using a voxelwise threshold of *z* = 2.3 and a cluster size probability of *p* < 0.05.

There were no significant effects in controls. ADHD subjects showed exclusive positive association between VN-AI and BOLD response across multiple extra-visual brain regions, the majority of which fell within the DMN. The ADHD-exclusive associations produced significant group differences (Table 7). To help interpret these findings in relation to extra-visual networks, activation maps are also presented with color-coded overlays that demarcate extra-visual network boundaries (Figure 5).

**Table 7:** **Visual network asymmetry association with BOLD signal during the letter task**.

			ADHD	*A* > *C*
	
Region	Hem	MNI	*Z*-val.	*Z*-val.
Frontal pole (lateral)	L	−28, 52, 34	3.6	2.5
Frontal pole (lateral)	R	46, 34, −8	3.8	2.9
Frontal pole (mid)	L	−10, 72, 6	3.5	3.7
Frontal pole (mid)	R	12, 64, 20	3.0	3.0
Superior-frontal gyrus (lat)	L	−20, 16, 46	3.6
Superior-frontal gyrus (mid)	Mid	4, 50, 36	3.0	2.4
Inferior frontal gyrus	R	48, 16, 16	3.4	3.7
Inferior frontal gyrus	R	58, 34, 10	3.6	3.0
Frontal operculum cortex	R	44, 16, 8	3.4	2.9
Frontal operculum cortex	R	46, 0, 14	3.7	3.9
Paracingulate gyrus	Mid	−2, 48, 14	3.1	2.9
Paracingulate gyrus	Mid-R	8, 48, 14	3.5	2.9
Pre-central gyrus (Inf)	R	62, 4, 14	3.2	
Post-central gyrus (Inf)	R	64, −10, 24	3.1	2.8
Post-central gyrus (Sup)	R	58, −10, 48	2.8	3.3
Temporal pole	R	46, 8, −40	3.0	2.8
Middle temporal gyrus (ant)	R	48, −2, −28	3.8	3.8
Middle temporal gyrus (Inf)	R	56, −28, −14	4.0	3.7
Inferior temporal gyrus	R	52, −28, −22	2.6	3.3
Middle temporal gyrus (post)	R	64, −22, −8	3.6	3.0
Temporal-occipital cortex	L	−66, −52, −8	3.9	3.3
Temporal-occipital cortex	R	60, −44, 6	3.0	2.7
Superior temporal gyrus	L	−64, −10, 6	4.3	3.0
Superior temporal gyrus (Lat)	R	62, −30, 2	3.4	3.2
Superior temporal gyrus (post)	L	−60, −40, 10	4.1	3.6
Superior temporal gyrus (post)	R	44, −34, 4	4.1	3.2
Angular gyrus	L	−62, −58, 30	4.0	3.5
Angular gyrus	R	62, −50, 28	3.7	3.9
Precuneus cortex	Mid-L	−6, −52, 46	4.4	
Precuneus cortex	Mid	4, −52, 48	3.5	
Superior parietal lobule	Mid-L	−6, −56, 66	4.1	
Superior parietal lobule	Mid	4, −56, 62	3.2	

*Table shows ADHD positive associations between visual network asymmetry (VN-AI) and extra-visual-network brain regions during the letter task. *A* > *C* indicates significantly greater positive association in ADHD versus controls. Results are ordered along anterior-to-posterior axis; Hem., hemisphere; L, left hemisphere; R, right hemisphere; Mid, activated voxel with *x*-coordinate between −5 and 5; MNI, Montreal Neurological Institute structural atlas coordinates (*x*-, *y*-, and *z*-axis); *Z*-val., *z*-value indicating BOLD signal intensity at reported voxel; Significance determined using a voxelwise threshold of *z* = 2.3 and a cluster size probability of *p* < 0.05*.

### Symptoms

These analyses are performed exclusively in ADHD subjects, as there was insufficient variability in symptoms metrics in controls to justify examination. Symptom data reflect DSM-IV criteria for inattentive and hyperactive subscales, obtained during K-SAD-PL semi-structured interviews (with mother and child participant). Due to the relative importance of symptom effects in ADHD, we have provided scatter plots to help guide interpretation of correlation findings.

#### ADHD symptoms association with VN-AI

Partial correlations (adjusted for age) indicated no relationship between symptoms and VN-AI during the letter–baseline condition.

#### ADHD symptoms association with extra-visual networks

Partial correlations (adjusted for age) indicated no relationship between symptoms and extra-visual networks during letter task (i.e., letter–baseline). However, trend level effects suggested possible associations between inattention and limbic (*r* = 0.39, *p* = 0.09), and default mode (*r* = 0.42, *p* = 0.06) network activation. During the location task, partial correlations (adjusted for age) indicated a positive association between inattentive symptoms and DMN activation (*r* = 0.51, *p* = 0.02) (i.e., more inattentive symptoms = more DMN activation), however, this effect did not survive Bonferroni correction for multiple testing (see Part 3 in Supplementary Material for scatter plot).

#### ADHD symptoms associations with voxelwise signal maps

**Figure 6:**
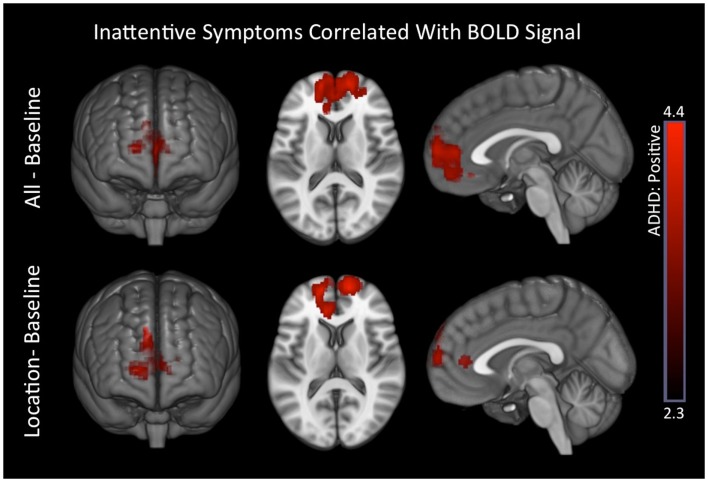
**Inattentive symptoms correlated with BOLD signal in ADHD subjects**. Positive associations between inattentive symptoms and BOLD responses in medial prefrontal regions that are associated with the DMN were observed in ADHD subjects for the all–baseline and location–baseline conditions. Images are thresholded using a voxelwise threshold of *z* = 2.3 and a cluster size probability of *p* < 0.05.

For all–baseline and location–baseline contrast, ADHD subjects exhibited several positive associations between inattentive symptoms and BOLD signal in medial prefrontal brain regions. There were no associations for hyperactive symptoms (Table 8; Figure 6). Also, see Part 3 in Supplementary Material for scatter plot of inattentive symptoms correlation to BOLD signal for above-threshold voxels depicted in the location–baseline condition.

**Table 8:** **Inattentive symptoms association with BOLD signal**.

			ADHD
Region	Hem	MNI	*Z*-val.
**ALL-BASELINE**			
Frontal pole	L	−14, 56, 8	3.70
Frontal pole	Mid	−4, 62, 8	3.51
Frontal pole	Mid-R	8, 58, 10	3.06
Frontal medial cortex	Mid	2, 48, 14	2.73
Paracingulate gyrus	L	−16, 46, −2	4.42
Paracingulate gyrus	Mid	0, 46, 8	3.33
Paracingulate gyrus	R	16, 54, 2	4.11
**LOCATION-BASELINE**			
Frontal pole	L	−14, 56, 10	4.06
Frontal pole	Mid	−4, 64, 10	2.90
Frontal pole	R	14, 60, 4	4.36
Frontal pole	R	10, 62, 30	3.08
Frontal medial cortex	L	−18, 48, −4	4.08
Cingulate gyrus (anterior)	Mid-L	−8, 36, 6	3.58

*Table shows ADHD positive associations between inattentive symptoms and BOLD response in all–baseline and location–baseline conditions. Hem., hemisphere; L, left hemisphere; R, right hemisphere; Mid, activated voxel with *x*-coordinate between −5 and 5; MNI, Montreal Neurological Institute structural atlas coordinates (*x*-, *y*-, and *z*-axis); *Z*-val., *z*-value indicating BOLD signal intensity at reported voxel; Significance determined using a voxelwise threshold of *z* = 2.3 and a cluster size probability of *p* < 0.05*.

#### Task performance association with VN-AI

Partial correlation analysis (adjusted for age) demonstrated that neither group showed any association between letter-task accuracy and VN-AI. Both groups showed non-significant associations between letter-task RT and VN-AI [ADHD (*r* = 0.24, *p* = 0.32), controls (*r* = −0.35, *p* = 0.13)]. Fisher’s *r*-to-*z* test indicated that the difference between the groups’ correlations was trending toward significance (*z* = 1.83, *p* = 0.067).

#### Task performance association with extra-visual networks

##### Letter–baseline

In ADHD subjects, letter-task performance was not significantly correlated with extra-visual networks. In controls, accuracy was negatively correlated with the limbic (*r* = −0.48, *p* = 0.03) and default mode (*r* = −0.57, *p* = 0.009) networks, but these effects did not survive adjustment for multiple testing (i.e., Bonferroni). Still, Fisher’s *r*-to-*z* test indicated they were different from ADHD subjects [LIM (*z* = 1.96, *p* = 0.05); DMN (*z* = 2, *p* = 0.045)]. Also, note that the DMN effect in controls was just shy of the Bonferroni corrected cut-off (*p* = 0.009 versus α = 0.008). In controls, letter-task RT was positively correlated with SOM activation (*r* = 0.45, *p* = 0.04), but this did not survive Bonferroni correction, and was not significantly different from ADHD subjects.

##### Location–baseline

In ADHD subjects, location-task performance was not significantly associated with extra-visual networks. In controls, accuracy was negatively correlated with DMN activation (*r* = −0.48, *p* = 0.03), but this effect did not survive Bonferroni correction, and was not significantly different from controls (Fisher’s *r*-to-*z* test: *z* = 1.68, *p* = 0.09).

#### Task performance association with ADHD symptoms

Partial correlations (adjusted for age) indicated no relationship between ADHD symptoms and behavioral performance.

#### Task performance association with voxelwise signal maps

##### Response time

Controls exhibited several positive associations between response time and BOLD signal in somatomotor brain regions. There were no group differences (see Part 4 in Supplementary Material for details).

##### Accuracy

**Figure 7:**
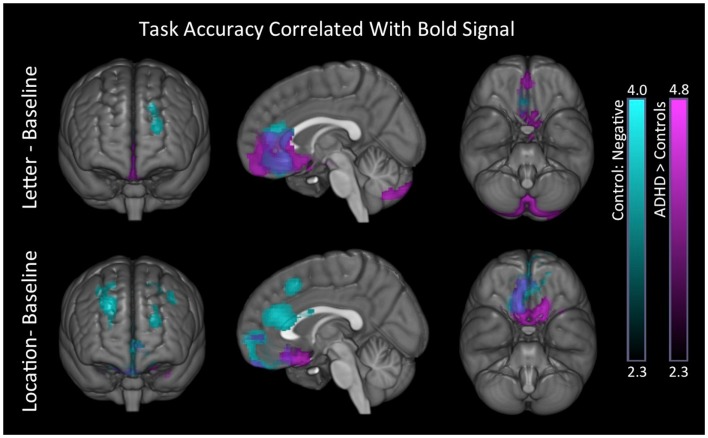
**Task behavioral accuracy association with BOLD signal**. In both conditions, controls exhibited negative associations between task accuracy and BOLD signal in superior-frontal and anterior medial brain regions (blue). We also found significant group differences in these same regions for the ADHD minus controls contrast (purple). Images are thresholded using a voxelwise threshold of *z* = 2.3 and a cluster size probability of *p* < 0.05.

Controls showed negative associations between tasks accuracy and BOLD signal in brain regions understood to reflect DMN activation. These associations also produced significant group differences (Table 9; Figure 7) for the ADHD minus controls contrast.

**Table 9:** **Task behavioral accuracy association with BOLD signal**.

			Controls	*A* > *C*
	
Region	Hem	MNI	*Z*-val.	*Z*-val.
**LETTER–BASELINE**				
Frontal pole	L	−18, 54, 40	−3.20	
Frontal pole	Mid	2, 54, −24		3.01
Frontal orbital cortex (inferior)	L	−10, 6, −20		3.83
Frontal medial cortex	R	10, 42, −16		3.31
Subcallosal cortex	Mid-L	−6, 28, −12	−3.45	3.42
Subcallosal cortex	Mid	4, 26, −14	−3.13	3.50
Cingulate gyrus (anterior)	Mid	0, 42, 8	−3.41	4.05
Paracingulate gyrus	L	−14, 38, 18	−3.24	
Occipital pole	L	−26, −100, −16		3.11
Cerebellum	L	−12, −80, −48		3.72
Cerebellum	Mid-R	6, −86, −42		3.43
**LOCATION–BASELINE**				
Frontal pole (inferior)	R	14, 36, −26	−4.16	4.47
Frontal pole (superior)	R	22, 44, 38	−3.95	
Frontal pole	Mid-L	−8, 64, −2	−3.37	2.94
Frontal pole	L	−22, 58, 24	−2.98	
Frontal orbital cortex	L	−12, 22, −24		4.05
Frontal orbital cortex	R	10, 30, −22	−4.88	4.71
Subcallosal cortex	Mid-R	8, 26, −22	−4.68	4.83
Middle frontal gyrus	L	−34, 24, 44	−3.28	
Superior-frontal gyrus	L	−18, 26, 52	−3.19	
Frontal medial cortex	Mid	−2, 52, −26	−3.02	2.74
Cingulate gyrus (anterior)	Mid	0, 28, 14	−3.60	
Cingulate gyrus (anterior)	Mid	2, 36, 20	−3.44	
Paracingulate gyrus (ant)	R	12, 38, 24	−3.36	
Paracingulate gyrus (dorsal)	Mid	2, 20, 48	−3.06	

*Table shows significant negative associations between tasks accuracy and BOLD response in letter–baseline and location–baseline conditions. A > C indicates significantly greater association in ADHD versus controls. Hem., hemisphere; L, left hemisphere; R, right hemisphere; Mid, activated voxel with *x*-coordinate between −5 and 5; MNI, Montreal Neurological Institute structural atlas coordinates (*x*-, *y*-, and *z*-axis); *Z*-val., *z*-value indicating BOLD signal intensity at reported voxel; Significance determined using a voxelwise threshold of *z* = 2.3 and a cluster size probability of *p* < 0.05*.

##### Post hoc analysis

For the purpose of data interpretation, two additional *post hoc* analyses were performed. Univariate ANOVA (adjusted for age) was used to examine whether there were group differences in the averaged DMN activation. There were no group differences in any task condition (*p* > 0.69). Partial correlations (adjusted for age) were used to examine association between location-task VN-AI and extra-visual networks (averaged signal) during the location task. There were none in either group.

## Discussion

The current study used recently developed fMRI methods to replicate and further examine identified abnormal rightward biased information processing in ADHD. Our task presented four-letter word stimuli and required subjects to detect a uniquely colored red letter and decide whether it was an “A” or not (letter task), or whether it was on the left or right (location task). Initial within-group analyses revealed a pattern of left-lateralized visual cortical activity in controls, but right-lateralized visual cortical activity in ADHD children. Our primary direct analyses of visual network asymmetry (VNA) confirmed that atypical rightward VNA was present in ADHD children and significantly different from controls in the letter task and overall. This finding adds to the growing literature that identifies abnormal information processing to be a key factor in ADHD. Moreover, in conjunction with our previous work (see Introduction section), it further specifies that ADHD abnormal information processing includes atypical increased weighting of RH versus LH contribution. Indeed, we have now demonstrated this characteristic using behavioral laterality (28, 29), EEG asymmetry (12, 31, 34), and here by examining asymmetry of fMRI BOLD signal.

Through our secondary aim, we additionally demonstrated that ADHD subjects’ rightward VNA during the letter task was atypically associated with reduced DMN activation. Recall that positive VNA scores reflect leftward asymmetry, with the reverse also true (i.e., negative VNA scores reflect rightward asymmetry). We found, in two separate analyses of BOLD signal in the letter task that ADHD subjects exhibited an atypical positive correlation between VNA and DMN signal. This indicates that leftward VNA is associated with greater DMN activation, and that rightward VNA is associated with reduced DMN activation. Given ADHD subjects’ atypical rightward VNA during the letter task, we focus our discussion of DMN findings on the link between rightward VNA and reduced DMN activation in ADHD. Regardless of the directionality, this and additional network findings importantly demonstrate that atypical rightward VNA in ADHD is associated with multiple distributed brain-systems, including previously implicated large-scale networks and frontal brain regions.

### Abnormal visual information processing and ADHD

Functional abnormalities in the visual cortex have proven to be a key feature of ADHD (2), with abnormal visual cortical structure identified (13, 117), and abnormal early-stage sensory information processing well established (14, 15, 26, 118, 119). This literature implicates ADHD deficits for both visual discriminations and categorization functions. The current study, along with our previously discussed findings (see Introduction), adds an important novel element to this topic – that is, abnormal information processing in ADHD involves atypical increased weighting of RH versus LH contribution to visual sensory information processing.

Hemispheric specialization of visual cortical functions notably includes LH specialization for linguistic stimuli and RH specialization for faces (for review see Ref. (120)). However, RH specialization is also reported for bottom-up functions, such as detection of sequence breaking novel objects (59), automatic assessment of object relevance (57), automatic perceptual/integrative category learning (58, 60), esthetic analysis (121), and within-category feature discrimination (122). Furthermore, RH specialization is well established for top-down task-directed attention functions, such as vigilance, sustained, and selective attention (123–128). Our current study finding of rightward VNA in ADHD suggests some form of increased weighting of these right-lateralized mechanisms.

#### Right-hemisphere contributions to VNA

The above noted right-lateralized brain functions reflect two classes of sensory information processing: self-directed top-down and automatic bottom-up. Within these domains, we can further distinguish processing that supports fast stimulus identification (i.e., categorization) versus in-depth sensory analysis. In the top-down domain, this reflects applied effort to identify/categorize a stimulus, or to scrutinize a stimulus’s details (129). In the bottom-up domain, this reflects mechanisms that automatically alert us to behaviorally relevant content in our surroundings, or that support fluid sensory-immersive experience (130, 131). In total, we conceptualize four variant domains of RH contribution to visual processing: (1) task-directed categorizations, (2) task-directed scrutiny of details, (3) bottom-up automatic categorizations, and (4) bottom-up sensory-immersive. A key premise of the current study, and our previous work, is that complex task-directed actions heavily rely on the first of these (i.e., task-directed categorizations) to support fast-efficient perceptual identification of task-stimuli, using the minimal sensory exposure required to do so. We refer to this as “task-specialized” sensory information processing, and conceptualize it to include varying mixtures of sustained, selective, and vigilance-related attentional functions, depending on the nature of a given task.

We have previously hypothesized (27) that any form of reduced ability for this task-specialized manner of visual information processing is likely to coincide with a proportional increased expression of non-task-specialized forms, resulting in greater possible expression of (a) unneeded scrutiny of visual details, (b) attentional shifting to off-task content, and/or (c) task-inappropriate orientation toward sensory-immersive processing. The net effect of this is expected to be an increased exposure to visual content beyond what is strictly required to perform task operations. This is conceptualized as “visual sensory overflow” in relation to task objectives. Our model postulates that this circumstance may underlie increased RH contribution to visual sensory processing in ADHD [for full model description see Ref. (27).

To examine this thesis, our current study was designed so that task conditions differentially engaged the task-specialized manner of visual information processing, but were otherwise perceptually identical. The letter task required subjects to identify a nominated target “A,” and distinguish it from other letters. This was expected to tax RH mechanisms that support top-down selective attention (123–128), which is a key aspect of task-specialized visual information processing. In contrast, the spatial condition did not require maintenance of a nominated target, or making categorical judgments about discrete items, and as such, was not expected to tax selective attention. Thus, to the extent rightward VNA in ADHD reflects reduced efficiency for task-specialized visual processing, we expected it to be maximally expressed during the letter condition. This supposition was born out. However, a non-significant pattern of rightward VNA in ADHD was also generally apparent. This suggests that in addition to an applied attentional effect underlying rightward VNA in ADHD, there may also be some form of default bias toward perceptual versus linguistic processing. This notion is further discussed below.

#### Left hemisphere contributions to VNA

The above discussion addresses possible sources of atypically increased RH contribution to visual processing, however, our current study did not, strictly speaking, uncover such an effect. We demonstrated increased rightward VNA in ADHD. This indicates a *relative* increased weighting of RH versus LH visual cortical contributions. An additional component of our proposed model (27) is that with optimal functioning of task-specialized visual information processing, efficient perceptual-level encoding of task-stimuli is expected to be quickly followed by the translation of perceptual content into verbal articulatory codes that facilitate updating of task-directives in verbal working memory (27, 132). According to this view, optimum performance of this system should be indexed by minimal resources having to be utilized at early perceptual stages. That is, information processing should move as efficiently as possible from perceptual-level to verbal categorization functions.

Consistent with this, both developmental and adult studies show a transfer of right to LH processing of visual information that coincides with the learning of new visual items and their name codes (133, 134). Ostensibly, with greater familiarity the requirement for perceptual-level analyses is reduced (likely due to greater use of predictive imagery), which allows faster transitioning from perceptual to verbal-categorical stages. Furthermore, recent work shows that transitioning into LH dominant modes of processing during linguistic operations is a function of RH inhibition rather than increased LH activation (135). These studies highlight that the relative efficiency of verbal sensory encoding is partly a function of earlier perceptual-level operations. In this vein, we suggest that the currently observed lack of normal leftward VNA in ADHD during the letter task, and associated worse accuracy, is likely a secondary consequence of abnormal perceptual stage processing. This view seems to also align with the identified slower naming speeds in ADHD, which occur absent any overt linguistic impairment (19–26). It is also consistent with our previous study that showed ADHD adults’ impaired ability for detecting word stimuli could be completely normalized by altering attentional parameters (29).

#### Abnormal left–right integration

Evidence of increased rightward VNA in ADHD is well aligned with identified reduced posterior corpus callosum size (41, 136) and abnormal function in ADHD (7, 42–44). In fact, the specific callosal region implicated in ADHD (the splenium) connects left and right visual cortices (137). This callosal region undergoes increase of myelination across development, resulting in greater interhemispheric EEG synchrony (particularly in alpha 8–12 Hz), and capacity to regulate lateralized visual cortical functions (138). Moreover, these changes include a progression from right-to-left dominance of visual cortical processing, and are suggested to reflect plastic tuning in response to childhood and adolescent maturation of visual ability (133, 134, 138). These findings suggest that abnormal VNA in ADHD might reflect some form of deviant maturation of posterior callosal functioning that bears on interhemispheric coordination of visual functions. Identified abnormal posterior EEG coherence in ADHD may be consistent with this view (7, 42, 43).

Another aspect of collosal functioning that is perhaps relevant to ADHD and our current finding has to do with the directionality of interhemipsheric transfer. Although previously considered symmetric, a recent study showed that a greater proportion of splenial collosal fibers project right-to-left than left-to-right (137). This is consistent with normally observed faster right-to-left callosal transfer times (44, 137). However, a study by Rolfe et al. (44) has indicated that ADHD subjects exhibit a reversed pattern of faster left-to-right transfer times (in combined types), and/or atypically slow right-to-left transfer (in inattentive types), suggesting a possible increased reliance on (or dominance of) RH visual cortical contribution. Moreover, a recent structural imaging study has reported larger RH visual cortical volumes in ADHD (13), and our previous laterality work demonstrated that ADHD adults are better able to inhibit pre-potent LH based stimulus responsivity than controls (28). Together, these studies support the view that there may be some form of default increased reliance on, or dominance of, RH contribution to visual sensory information processing in ADHD. If true, this default or state-independent aspect may function as a separate but additive factor to the more applied attentional effects previously discussed.

### Visual network asymmetry and extra-visual networks

Our previous work indicated that atypical rightward asymmetry in ADHD is sensitive to top-down modulation of attention and brain-state orientation (11, 28, 29), and others have shown that ADHD cognitive impairments can be sensitive to alterations in motivation (139). However, findings of structural and functional deviations at rest also clearly implicate more fixed or state-independent abnormal brain function in ADHD (136, 140, 141). In an attempt to further clarify the nature of abnormal brain function underlying rightward VNA in ADHD, the current study examined the association between VNA and extra-visual networks, several of which are implicated in ADHD (2, 3). In particular, our aim was to examine the association between rightward VNA and DMN function.

Default mode network function has been widely investigated in recent years, with multiple studies linking it to ADHD (2, 3). Although previously characterized as a resting or task-negative network (65), studies have now indicated an active role in internally directed self-referential aspects of cognition, also highlighting that its interactive dynamics with other networks are more flexible and circumstance-specific than previously understood (66, 68, 74, 142). To this end, Wang et al. (68) showed that most aspects of the DMN increased coherence during a word-picture matching task, with the lone exception being connectivity between bilateral posterior cingulum and RH inferior parietal cortex. They suggested greater on-task coherence of DMN reflects internal task-mediating processes, and concluded that DMN function is engaged during tasks, but in specific fashions rather than absolutely suppressed. Hampson et al. (67) were an early proponent of a similar view, suggesting DMN function is engaged during cognitive challenges to facilitate or monitor cognitive performance. Furthermore, Uddin et al. (74), using Granger causality analysis, have demonstrated DMN direct modulation of task-positive networks. Finally, a recent study by Yakushev et al. (69) has shown a link between DMN integrity and verbal working memory ability. Together, these findings raise the intriguing possibility that abnormal DMN function in ADHD might be associated with a reduced capacity to orchestrate the internal aspects of task-directed cognition (e.g., planning, initiating, maintaining, and updating task-directives) (70–73). If true, this could underlie a general reduced capacity for task-directed brain functions in ADHD, including poor task-directed visual sensory information processing.

The current study showed that VNA in ADHD was more generally and robustly associated with extra-visual networks compared to controls. The generality of these associations may fit with the above view insomuch as abnormal DMN function in ADHD might be synonymous with having a less stable task-directed neural architecture (27, 143), with associated poorer on-task modulation of task-positive networks. This suggests that ADHD task-directed brain functioning may generally occur in a less coordinated or piecemeal manner. If true, ADHD subjects may have needed to more often adjust effort between component “internal verbally weighted,” and “external perceptually weighted” operations, resulting in a greater general association between VNA and extra-visual networks during our letter task.

In addition to the above noted general effects, a critical role for DMN function in ADHD was also directly indicated. VNA association with DMN signal was one of the three effects that survived Bonferroni correction for multiple testing. Moreover, DMN signal showed unique abnormal association to both inattentive symptoms and behavioral performance in ADHD subjects. Inattentive symptoms showed a positive association with medial anterior aspects, while ADHD subjects showed no behavioral association with DMN function, with controls exhibiting the expected pattern of greater accuracy with reduced DMN activation (also involving medial anterior aspects). Moreover, and consistent with the above discussion, these effects occurred mainly during the more difficult letter-task condition, which ostensibly placed greater demands on internal task processing, possibly including an increased requirement for DMN modulation of task-positive networks (74).

With regard to the directionality of effects, our findings showed a pattern of positive association between the VN-AI metric and all extra-visual networks examined. This means that among ADHD subjects leftward VNA was associated with stronger network signal, while rightward VNA was associated with reduced network signal. Given our primary finding of increased rightward VNA in ADHD, the latter aspect is most relevant. That is, atypical increased rightward VNA in ADHD during the letter task was associated with reduced network signal, most notably for the default mode and VAN. Reduced DMN activation occurs with active externally oriented processing (66). Reduced VAN activation has been linked to having a fixed or stable attentional set (57–60). This suggests that rightward VNA in ADHD during letter discriminations may reflect some form of externally oriented task-adaptive or compensatory processing. This speculation is supported by the trend effect showing ADHD subjects were faster with greater rightward asymmetry (and slower with leftward asymmetry), while controls showed an opposite pattern. Moreover, ADHD subjects exhibited a unique and robust speed–accuracy tradeoff during the letter task, which may be consistent with effortful compensatory processing.

#### Additional considerations

As noted, medial prefrontal aspects of the DMN network were associated with ADHD inattentive symptoms and task performance. This brain region has been identified as a source of top-down regulation of the brain-stem locus coeruleus (64), which via dense noradrenergic projections to the RH is critical for managing transitions between controlled and flexible attention and cognitive sets (144, 145). Moreover, noradrenergic projections via the locus coeruleus have also been shown to play an active role in modulating RH visual cortical functions (146). Given abnormal norepinephrine (147), DMN (3), and RH visual cortical volume in ADHD (13), the above noted circuits suggest a possible mechanism by which abnormal DMN function may be linked to both dysregulated attention-state setting (e.g., exploratory versus task-oriented) and greater RH visual cortical contribution in ADHD.

Default mode network influence over applied attention may also occur. As noted, Wang et al. (68) reported that most aspects of DMN circuitry increased coherence during a word–picture matching task, with an exception being connectivity between bilateral posterior cingulum and the RH inferior parietal cortex. Franzen et al. (148) reported reduced resting-state DMN connectivity in ADHD subjects between this same DMN posterior cingulum region and RH inferior parietal cortex. Given the well-established role of RH inferior parietal cortex in top-down attentional functions [for review see Ref. (149)], it is interesting to consider that this aspect of DMN circuitry might somehow undermine ADHD subjects’ ability to direct task-specialized visual sensory information processing. Finally, the possibility that abnormal DMN function impacts both attention-state and applied attention mechanisms in ADHD may be consistent with findings presented by Uddin et al. (74) showing bipartite DMN functions, with differential impacts on RH inferior parietal and anterior regions.

## Conclusion

The current study demonstrated rightward VNA in ADHD during a simple letter discrimination task. This result, in conjunction with our previous findings, adds an important novel consideration to the growing literature identifying abnormal visual sensory information processing in ADHD. We expect rightward VNA reflects increased perceptual engagement of task-extraneous content, and that this occurs with any form of reduced ability for top-down task-directed visual sensory information processing. The current study also identified that rightward VNA in ADHD was atypically and robustly associated with multiple extra-visual network systems, namely the DMN and VAN. Rightward VNA in ADHD was associated with reduced activation in these networks, possibly indicating some form of task-adaptive compensatory processing. Moreover, we also identified abnormal DMN associations with ADHD inattentive symptoms and behavioral performance during our letter task. We postulate that abnormal DMN function in ADHD may index a general reduced capacity to induce and/or maintain a task-adaptive neural architecture, with negative cascading effects resulting in less efficient task-directed perceptual encoding of visual stimuli, and associated increased rightward VNA.

## Conflict of Interest Statement

The authors declare that the research was conducted in the absence of any commercial or financial relationships that could be construed as a potential conflict of interest.

## Supplementary Material

The Supplementary Material for this article can be found online at http://www.frontiersin.org/Journal/10.3389/fpsyt.2014.00081/abstract
