# Supplementary material for: Visual Network Asymmetry and Default Mode Network Function in ADHD: An fMRI Study
Source: Front Psychiatry. 2014 Jul 15;5:81. doi: 10.3389/fpsyt.2014.00081 (PMC4097354; doi:10.3389/fpsyt.2014.00081)
Supplement: Supplementary file 1 [file Data_Sheet1.DOCX]

**Supplement**

**Visual Network Asymmetry and Default Mode Network Function in ADHD:**

**An fMRI Study**

**Part 1: Supplement Figure 1: Task Graphic**

**
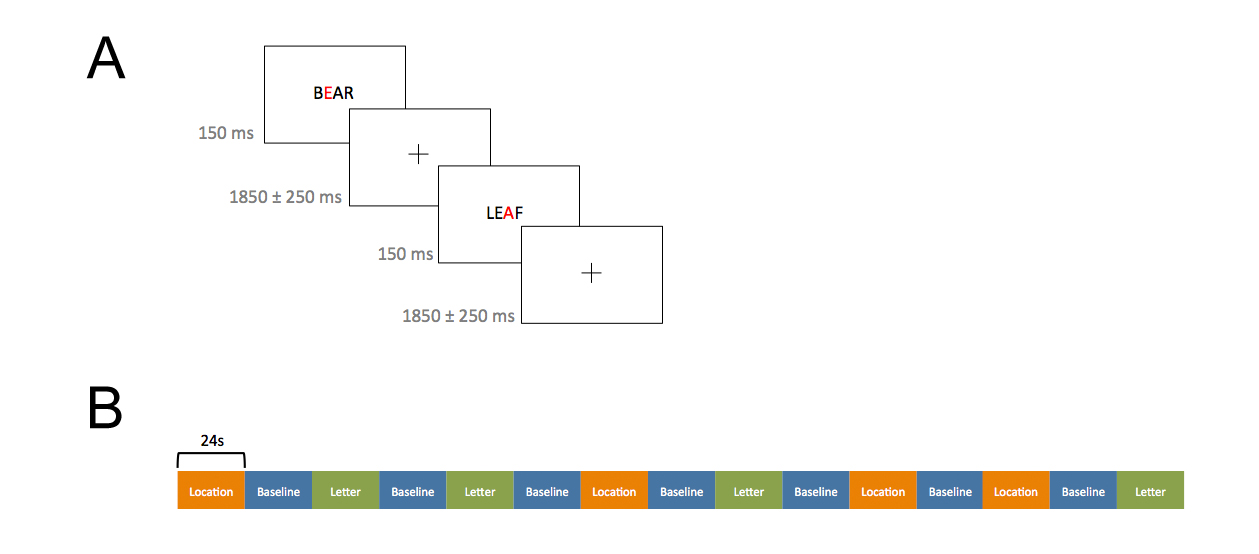
**

Supplement Figure 1:  Task paradigm.  A) Each trial lasted about 2 seconds, with ±250ms of jitter. In the location task, subjects decide if the red letter is an ‘A’ or not.  In the location task, subjects decide if the red letter is on the right or left of center. In the baseline task, subjects made no decision but pressed a button in response to every stimulus.  B) Subjects completed blocks of 12 trials lasting 24 seconds each.  Each scan consisted of 4 blocks of the location task and 4 blocks of the letter task, with the baseline task interspersed.

**Part 2: Task Comparison**

Supplement Table-1 provides detailed findings from Location-Letter contrast. ADHD and control subjects exhibited several overlapping activations in brain regions thought to be linked to DMN activity. Additional unique activations were evident in subcortical regions among ADHD subjects, and within somatomotor regions among in controls. Direct comparison between the groups did not show significant differences.

**Supplement Table 1: Location – Letter: Within Group Effects**

| **Location-Letter** |  |  | **Control** | **ADHD** |
| --- | --- | --- | --- | --- |
| Region | Hem. | MNI | Z-Val. | Z-Val. |
| Common |  |  |  |  |
| Frontal Pole | L | -12, 62, 4 | 5.13 | 5.54 |
| Frontal Pole | R | 12, 46, 48 | 3.54 | 3.13 |
| Superior Frontal Gyrus | L | -20, 32, 42 | 4.7 | 4.8 |
| Middle Frontal Gyrus | L | -32, 28, 52 | 3.45 | 3.52 |
| Orbital Frontal Cortex | L | -22, 26, -24 | 3.0 | 3.0 |
| Orbital Frontal Cortex | R | 38, 26, -18 | 3.14 | 3.18 |
| Temporal Pole | L | -34, 6, -22 | 3.21 | 3.27 |
| Temporal Pole | R | 36, 24, -28 | 4.2 | 3.18 |
| Middle Temporal Gyrus (Ant) | L | -64, -32, -18 | 4.02 | 3.34 |
| Middle Temporal Gyrus (Post) | L | -56, -58, 2 | 3.8 | 3.1 |
| Middle Temporal Gyrus (Post) | R | 54, -54, 2 | 4.2 | 3.5 |
| Lateral-Occ/Inferior-Parietal | L | -44, -70, 26 | 4.15 | 3.63 |
| Lateral-Occ/Inferior-Parietal | R | 44, -70, 26 | 3.72 | 3.76 |
| Parahippocampal Gyrus | L | -26, -34, -16 | 2.93 | 3.56 |
| Hippocampus | L | -20, -14, -32 | 2.8 | 2.9 |
| Paracingulate Gyrus | Mid | 0, 46, 0 | 3.8 | 4.1 |
| Cingulate Cortex (Anterior) | Mid | 0, 42, 14 | 3.0 | 4.72 |
| Cingulate Cortex (Posterior) | Mid | 0, -30, 38 | 3.7 | 3.13 |
| Subcallosal Cortex | Mid | 0, 18, -20 | 2.83 | 3.34 |
| Precuneus | Mid | 0, -58, 24 | 3.38 | 3.5 |
| Lingual Gyrus | Mid | 0, -86, -8 | 4.42 | 3.1 |
| ADHD Only |  |  |  |  |
| Amygdala | L | -18, -6, -12 |  | 2.9 |
| Pallidum | R | 14, 2, -2 |  | 3.43 |
| Thalamus | Mid-R | 8, -8, 8 |  | 2.7 |
| Brain Stem | Mid | -2, -26, -26 |  | 3.42 |
| Controls Only |  |  |  |  |
| Frontal Pole (lateral) | R | 48, 44, -4 | 3.14 |  |
| Middle Frontal Gyrus | R | 48, 16, 38 | 3.62 |  |
| Precentral Gyrus | R | 58, 8, 18 | 3.6 |  |
| Middle Temporal Gyrus (Ant) | R | 60, -4, -30 | 3.1 |  |
| Middle Temporal Gyrus (Mid) | R | 66, -22, -20 | 3.39 |  |
| Auditory Cortex | R | 62, -4, 4 | 3.83 |  |
| Audiotry Cortex | L | -56, -4, 0 | 3.23 |  |
| Superior Parietal Lobule | R | 36, -48, 62 | 3.3 |  |
| Supramarginal Gyrus | R | 62, -24, 40 | 3.3 |  |
| Parietal Operculum | L | -52, -30, 26 | 3.1 |  |
| Angular Gyrus | R | 62, -50, 24 | 3.3 |  |
| Hippocampus | R | 30, -22, -12 | 3.43 |  |
| Amygdala | R | 26, 0, -18 | 3.3 |  |
| Lateral-Occipital Cortex | R | 40, -84, -16 | 3.3 |  |
| Insular Cortex | L | -40, -6, -4 | 2.8 |  |
| Cerebellar Crus I | R | 48, -66, -40 | 3.94 |  |
| Cerebellar Crus I | L | -50, -65, -36 | 2.92 |  |
| Cerebellar Crus II | R | 14, -84, -38 | 3.44 |  |
| Cerebellar Crus II | L | -18, -84, -38 | 3.15 |  |

Table shows significant within group activations for Location-Letter condition ordered along anterior-to-posterior axis. Table is organized to show: common, ADHD exclusive, and control exclusive findings. **Hem.**= hemisphere; **L**= left hemisphere; **R**= right hemisphere; **Mid**= activated voxel with x-coordinate < |5|; **MNI**= Montreal Neurological Institute structural atlas coordinates (x, y, and z axis); **Z-Val**.= z-value indicating bold signal intensity at reported voxel (see methods for details- all reported voxels significant at the cluster level).

**Part 3: Scatter Plots of Correlations Between ADHD Inattentive Symptoms and BOLD Response**

Due to the relative importance of symptom effects in ADHD, we’ve provided scatter plots to help guide interpretation of correlation findings.  Below we show scatter plots for two findings derived from the location task condition.

**Supplement Figure 2: ADHD Subjects’ Inattentive Symptoms Correlation with Averaged Default Mode Network Activation**

**
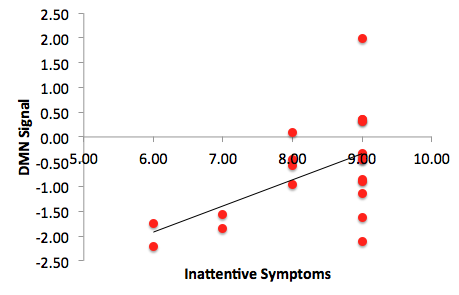
**

Scatter plot shows partial correlations (adjusted for age), showing a positive association between inattentive symptoms and averaged DMN activation during the location task (r=.51, p=.02). This effect did not survive Bonferroni correction for multiple testing.

**Supplement Figure 3: ADHD Subjects’ Inattentive Symptoms Correlation with Above-threshold voxels During Location Task**

**
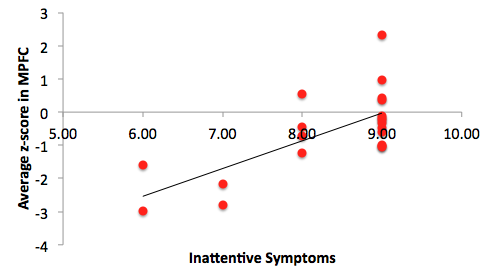
**

Scatter plot shows correlations between averaged Z-score of above threshold voxels and ADHD subjects’ inattentive symptoms during the location task. Above threshold voxels occurred exclusively within the medial prefrontal cortex (MPFC). Images are thresholded using a voxelwise threshold of z = 2.3 and a cluster size probability of p < .05.

**Part 4: Behavior (Response Time) Association to Voxelwise BOLD Response**

Supplement Table 2 provides details of the analysis to examine association between response time (RT) during the letter task and voxelwise signal maps. Controls exhibited several positive associations between response time and bold response in somatomotor brain regions. There were no group differences.

**Supplment Table 2: Task Response Time Association with Bold Signal**

| **Reaction Time**  **Correlated With Bold Signal** |  |  |  |
| --- | --- | --- | --- |
|  |  |  | **Controls** |
| Region | Hem | MNI | Z-Val. |
| **Letter-Baseline** |  |  |  |
| Precentral Gyrus | L | -14, -18, 72 | 2.89 |
| Precentral Gyrus | R | 24, -24, 72 | 3.12 |
| Postcentral Gyrus | L | -34, -34, 70 | 2.77 |
| Postcentral Gyrus | R | 50, -12, 58 | 3.72 |
| Postcentral Gyrus (Superior) | R | 42, -28, 70 | 3.44 |
| Supplementary Motor Cortex | Mid | -4, -4, 62 | 3.17 |
| **Location-Baseline** |  |  |  |
| Superior Frontal Gyrus | R | 18, 12, 64 | 3.10 |
| Middle Frontal Gyrus | R | 34, 10, 54 | 2.70 |
| Supplementary Motor Cortex | Mid | -4, -14, 54 | 3.34 |
| Supplementary Motor Cortex | R | 10, -4, 52 | 3.27 |
| Precentral Gyrus | L | -42, -12, 48 | 3.48 |
| Precentral Gyrus | R | 12, -22, 48 | 2.63 |
| Postcentral Gyrus | L | -42, -22, 44 | 3.97 |
| Supramarginal Gyrus | L | -56, -40, 48 | 2.85 |

Table shows significant associations between tasks response time and bold response in letter-baseline and location-baseline conditions. For each group (Controls, ADHD) any positive (Pos.) or negative (Neg.) correlations are shown, as well as any group differences indicating greater positive association in control (C-A) or ADHD subjects (A-C). **Hem.**= hemisphere; **L**= left hemisphere; **R**= right hemisphere; **Mid**= activated voxel with x-coordinate < |5|; **MNI**= Montreal Neurological Institute structural atlas coordinates (x, y, and z axis); **Z-Val**.= z-value indicating bold signal intensity at reported voxel (see methods for details- all reported voxels significant at the cluster level).
